# The Effects of Activating the Money Concept on Perseverance and the Preference for Delayed Gratification in Children

**DOI:** 10.3389/fpsyg.2016.00609

**Published:** 2016-04-27

**Authors:** Agata Trzcińska, Katarzyna Sekścińska

**Affiliations:** ^1^Faculty of Psychology, University of WarsawWarsaw, Poland; ^2^Institute for Social Sciences, University of WarsawWarsaw, Poland

**Keywords:** money activation, economic socialization, perseverance, delaying gratification, symbolic money

## Abstract

The psychological model of thinking about money assumes that implicit reminders of money lead to self-sufficient motivation. Previous research has demonstrated that children react to money in similar ways to adults. The priming of young children with money related concepts or images has negatively affected their social behavior and social preferences, leading them to make more individualist and less pro-social choices and be less willing to help others. The aim of this research was to investigate the positive influence of money activation on children’s behavior. The participants were 6–8 year old children who do not yet fully understand the instrumental function of money due to their young age. Two experimental studies were performed, the first of which analyzed the effect of perseverance and performance on a challenging task and the second investigated preferences with respect to delaying gratification. Sixty-one children aged 6 took part in the first study and forty-six scout camp participants 6–8 years of age were involved in the second experiment. The results support the hypotheses concerning the effects of money activation stating that (1) money activation influences children’s perseverance and effectiveness in difficult individual tasks, and that (2) it increases children’s preferences for delayed gratification. These results suggest that money has a symbolic power which may exert both positive and negative effects on children’s behavior. Since children between the ages of 6 and 8 do not understand the instrumental function of money fully, certain symbolic meanings of money may have been responsible for the money priming effects. The findings suggest that the symbolic function of money is more primal than its instrumental function and that it probably develops at an earlier stage in life.

## Introduction

One of the models of the psychological consequences of thinking about money that was developed recently (Vohs et al., 2006, 2008) assumes that money, being the most common form of reward, directs our attention toward personal contribution and profits, and leads to self-sufficient motivation, which is reflected in greater efforts to achieve personal goals and an increased preference for isolating oneself from others. Studies conducted by Vohs et al. (2006, 2008) have shown that subliminal priming with money-related concepts or images makes respondents: (1) less willing to help others or to donate money; (2) less likely to seek help with difficult or insoluble problems; (3) exert more effort to attaining personal goals; (4) prefer working and playing alone; and (5) establish a greater physical distance between themselves and other people. A number of studies that were conducted by other researchers have demonstrated alternate consequences of money activation like a sense of power, self-confidence and effectiveness; a tendency to exploit other people; the holding of liberal social attitudes and an increased need for self-determination (Gino and Pierce, 2009; Zhou et al., 2009; Liu et al., 2012; Caruso et al.’s, 2013; Yang et al., 2013). In summary, recent studies have shown that thinking about money can have both negative (e.g., lower willingness to help others), and positive (e.g., higher persistence in personal goals) consequences (Mogilner, 2010). Money priming’s desirable performance-related outcomes have been corroborated in experiments carried out over a wide range of locations (e.g., North America, Europe, and Asia) (Vohs, 2015). Similar effects seem to be exhibited by people of different ages (e.g., college students and adults) and by variant methods of priming money (e.g., viewing images of money, touching money, or seeing play money). Experiments that have been conducted on the positive effects that money priming has on performance have demonstrated that people put more effort into goal-oriented tasks (Vohs, 2015) after money activation and exhibit higher task persistence by spending more time on a difficult task than people primed with neutral concepts (Mogilner, 2010; Vohs, 2015). Mukherjee et al. (2013) also revealed that money priming increases a sense of self-efficacy. Greater self-efficacy and task persistence are probably the reasons behind the higher performance in different tasks after money concept activation, which was confirmed in various experiments (Boucher and Kofos, 2012; Vohs, 2015). Real money can serve as a powerful incentive for people to persist in effortful tasks by instilling motivation (Muraven and Slessareva, 2003). However, as mentioned earlier, money priming alone, apart from actual money, is already enough to produce the same effect. The very act of thinking about money makes people feel efficacious, capable and confident and, therefore, empowered to persevere (Boucher and Kofos, 2012).

All the results of aforementioned research studies reflect the symbolic or affective connotations of money rather than its instrumental effects. The distinction between the symbolic and instrumental meaning of money shows that money can be perceived as a tool for economic transactions (instrumental meaning) but its mental representation can also be full of symbolic and emotional meanings (symbolic meaning). Money, which was developed many years ago as a useful means to achieving aims (instrumental function), has acquired numerous cultural associations and meanings. Research that takes both the instrumental and symbolic functions of money into account has been undertaken in various disciplines. Psychologists, anthropologists and sociologists have all analyzed the social nature and social or psychological significance of money. They have argued that money has great symbolic and social meaning apart from its economic exchange function. The theory of “sacrum and profanum” (Belk and Wallendorf, 1990) posits that consumer societies worship money and attach various emotional meanings to it. Lea and Webley’s (2006) “tool/drug” motivational theory of money also asserts that money possesses a dual nature wherein, on the one hand, it is a means of purchasing valued goods, services or experiences and, on the other, it can be perceived as being intrinsically attractive or valuable. The contrast between the symbolic and instrumental functions of money has been reflected in research on money attitudes (Yamauchi and Templer, 1982; Furnham, 1984; Gąsiorowska, 2013). There are also numerous studies indicating that the social meaning of money may influence interpersonal and intrapersonal behavior, human motivation and human welfare alike (e.g., Trachtman, 1999; Zhang, 2009; Zhou et al., 2009).

Most research on the symbolic and instrumental functions of money has involved adults. Economic psychologists agree, however, that our understanding of money is shaped by the process of economic socialization that begins in early childhood. In the 1980s, systematic efforts were made to describe the process by which children acquire economic knowledge on the basis of Piaget’s theory of cognitive development (Piaget and Inhelder, 1972) and the assumption that the stages of economic knowledge acquisition are related to the stages of cognitive development. The proposed number of stages varied from three (Jahoda, 1979; Burris, 1983; Leiser, 1983), to ten (Strauss, 1952), Furnham and Argyle (1998), however, noted in more recent studies that this trend combines the sub-stages to form three main phases: (1) no understanding at all, (2) understanding of some isolated concepts, and (3) linking of isolated concepts to achieve a full understanding. The first phase corresponds to the preoperative stage of cognitive development. By 4–5 years old, children know that money is used in a shop but they still think that one coin buys one thing only and they do not understand the divisibility of money; shopping is a kind of ritual for them – they understand the general idea of money but they do not recognize its nominal value (Berti and Bombi, 1988). The second phase of economic development corresponds to the Piagetian concrete operations stage. According to Piaget’s theory, it lasts from the age of 5–6 years until eleven or 12 years old and is a time when children learn arithmetic operations and acquire the ability to consider the multiple features of an object at the same time. However, they still do not possess an understanding of the complex network of economic dependencies. Children finally start to integrate the concepts of buying and selling and grasp the idea of profit and investment on the next stage of cognitive development, at about the age of 11 (Piaget’s formal operations stage).

The research mentioned above has addressed the developmental changes in the understanding of the instrumental (not symbolic) function of money, which covers a variety of different aspects. These include, for example, an awareness of the purpose that money serves, the ability to calculate prices and change and an awareness of the purchasing power of money. The studies of Berti and Bombi (1988) that were conducted on a sample of 100 children in Italy as well as those of Kupisiewicz (2004), which included 700 children in Poland, demonstrated that the formation of an instrumental understanding of money is a process. The understanding of money undergoes many changes during the second phase of economic development. Children are aware of what money can be used for from the age of 4–5. In the studies of both Berti and Bombi (1988) and Kupisiewicz (2004), when 4- to 5-year- old children were asked what money serves for, they answered that it is used “to pay” or “to purchase things”. Thus, children already possess some instrumental understanding of money at this age. To describe the further developmental changes in children’s instrumental knowledge about money, Kupisiewicz (2004) studied Polish children on the second, general level of economic socialization (5–9 years of age). Her results have indicated that children between the ages of 5 and 6 think that the size of the note is connected with its value (i.e., a bigger banknote or coin is more valuable). Children at this age also believe that a banknote is always considerably greater in value than a coin. Children also do not understand the concept of changing money and perceive it as exchanging money for something essentially similar. The results of Kupisiewicz’s studies (ibidem) also revealed that when children are approximately 7 years old they learn to recognize different nominal values and are able to tender the exact amount of money in a shop. At this age, children begin to associate the value of money with the denomination and not with its appearance, although they are still incapable of correctly comparing the value of a few coins and one banknote. They are just beginning to grasp what changing money is about but still make mistakes when trying to exchange one banknote for a few coins, as they are still mainly influenced by the size and the gilding of coins. At the age of 8, a significant number of children still have problems with identifying coin and note denominations and have problems with giving the right change in a monetary transaction. Nine-year-olds are already familiar with the fact that the denomination reflects the value of money and that banknotes have a greater nominal value than coins. They are also capable of ascertaining the equivalence between banknotes and a few coins and can efficiently change coins and banknotes (Kupisiewicz, 2004).

To sum up, the studies conducted by Kupisiewicz (2004) indicate that children between 6 and 8 years of age have some, but not a full instrumental understanding of money. They understand the main function of money, they can recognize different nominal notes and coins but are still incapable of calculating the right prices and change and are unfamiliar with the prices of products and services. Basing on the results of Kupisiewicz (2004), Gąsiorowska et al. (2012) stated that children understand the instrumental function of money once they are capable of using it properly in economic transactions (i.e., they possess sufficient mathematical skills to know exactly how much should be paid for goods and are familiar with coins and notes and understand the notion of giving change). According to them, in the second, general phase of economic development children under the age of 8 do not fully understand the instrumental meaning of money yet.

The research presented above concerning children’s understanding of money has addressed the developmental changes in the process of gaining an understanding of the instrumental function of money, however, there is a dearth of research on how symbolic perceptions of money develop during economic socialization. Research has already provided exact knowledge of when children start to recognize coins and notes, understand the concept of giving change and correctly calculate prices (Kupisiewicz, 2004), but there still is a lack of knowledge about children’s understanding of the symbolic meaning of money. It still remains to be exhaustively explained whether or not the symbolic understanding of money comes with instrumental knowledge about it or if it develops independently. To the best of our knowledge, the only study that has addressed this issue so far was that of Gąsiorowska et al. (2012), wherein children in the second, general phase of economic socialization (in accordance with Piaget’s theory and the research conducted by Kupisiewicz (2004) who are still incapable of using money properly in economic transactions (i.e., do not fully understand the instrumental function of money) were examined in greater detail. They expected children at this stage of economic development to be capable of reacting to the symbolic meaning of money despite not yet having a full understanding its instrumental function. Gąsiorowska et al. (2012) conducted two experiments with 5 to 8-year-old participants based on Vohs’s model of the psychological consequences of money, wherein they considered the various symbolic meanings of money. They showed that children react to money in a similar way to the adults in the studies conducted by Vohs et al. (2006, 2008). After being reminded of the concept of money (money activation), children made more selfish choices in economic games, reported less pro-social preferences, and were less willing to help the experimenter than the children from the control group. Taking into account that the children participating in the experiments did not have a full understanding of the instrumental function of money, the authors concluded that children may acquire an understanding of the symbolic meaning of money before they actually understand its instrumental function. The influence of money activation on children’s behaviors was confirmed in subsequent research (Gąsiorowska et al., 2016), which proved that handling money (compared with other objects) reduced helpfulness and generosity in children. The authors interpreted their findings by suggesting that thinking about money inhibits communal goals in children.

Gąsiorowska et al. (2012) conducted research aimed at examining whether or not young children who still do not have the proper understanding of the instrumental function of money are susceptible to the activation of the symbolic meanings of money. The results obtained highlighted the importance of distinguishing between the development of an understanding of the instrumental and symbolic functions of money (ibidem). This initial study revealed the negative effects of thinking about the symbolic meaning of money (more individualist choices and a reduced willingness to help others) but it failed to address the issue of whether or not thinking about money might also have positive consequences in children, such as increasing perseverance and the length of time individuals are prepared to spend on their work. Studies on adults and undergraduates (Vohs et al., 2006, 2008; Mogilner, 2010; Vohs, 2015) have shown that money priming can induce some desirable performance-related outcomes but that there still is a deficit of studies verifying the effect of money priming on children’s positive behaviors. Taking into account that Gąsiorowska et al. (2012) study only shows the “dark” side of the symbolic meaning of money, the research reported herein intends to follow these experiments up. The main goal of the current study is to investigate the positive, behavioral effects of money activation on children. Following the previous experiments (ibidem), we decided to study children up to the age of 8 who, due to their age, do not fully understand the instrumental functions of money yet. To strengthen this assumption, a pre-test study was conducted in which the instrumental knowledge of money among children between the ages of 6 and 8 was verified. Taking these results (and the results obtained by Kupisiewicz, 2004) into account, we expected the susceptibility to money priming to mainly reflect the symbolic meanings of money. Two hypotheses, based on the idea that the symbolic meaning attached to money determines its influence on human behavior, were formulated and tested.

## Pre-Test Study

The main goal of the pre-test study was to verify the instrumental knowledge of money among children between 6 and 8 years of age. The study was conducted to confirm the results obtained by Kupisiewicz (2004), which constituted the basis for our assumptions stating that children between 6 and 8 years of age do not possess a comprehensive understanding of the instrumental function of money.

### Materials and Methods

#### Participants

Twenty-four children (12 girls and 12 boys) aged 6–8 years took part in the study (*M* = 7.00, *SD* = 0.78). The research was carried out in one primary school in Warsaw, Poland. Parents of all the children provided written informed consent for their children’s participation in the study.

#### Procedure

In our pre-test study, the methods designed by Kupisiewicz (2004) were implemented. All the children participating in the study were asked to answer several questions and carry out specific tasks involving money. At the beginning, an experimenter showed some Polish coins and notes to the children and asked them two questions: “What are these items?”, and “What can you do with them?” Next, the experimenter placed all the available Polish coins (unordered) in front of the child and asked the participant to indicate different denominations (1, 2, 5, 10, 20, 50 gr, 1PLN, 2PLN, 5PLN). A similar task involved Polish banknotes (10PLN, 20PLN, 50PLN, 100PLN). Another task was to differentiate Polish coins and notes from other presented objects. The experimenter then placed the binding Polish money in front of the child along with other tokens, buttons, and money from the Monopoly game as well as old Polish money that was withdrawn from circulation in 1997 [after the denomination in Poland when 10,000 old Polish zloty (PLZ) became one new Polish zloty (PLN)]. The child was then asked to indicate which items are now used to pay for and buy things in Poland. The next task was to identify which Polish coins (from the presented set) had the greatest purchasing power. Children viewed the set of unordered coins (from 1 gr to 2PLN) and were asked to indicate the coin with which they could buy the biggest amount of sweets. After this, the experimenter arranged coins ranging from 1 gr to 2PLN (four coins of each denomination) in front of the child and asked the participant to change the 5PLN coin by selecting coins from the set presented in front of the child. The last two tasks included calculating the exact price of something and giving change. Children were asked to give the experimenter 7PLN, 21 gr using the coins in front of them (four coins of each denomination, ranging from 1 gr to 2PLN, were placed in front of the participants). Finally, the children were told that the experimenter now wants to give them 7PLN, 21 gr, but has no coins, only a 10PLN banknote. The children were then asked what should be done in the situation when the experimenter gives them more money than required and, if they responded that they should give change, they were asked to give the experimenter the appropriate coins (from the set of four coins from each denomination, ranging from 1 gr to 2PLN).

### Results

Only one child responded correctly to all the questions asked by the experimenter but each of the above-mentioned tasks had different levels of difficulty for the participants. Almost all the children answered the first question correctly by saying that the items that were presented to them constituted money. Only one girl said that she had “forgotten the name”, but she knew what it was used for (and correctly answered the second question). Two older boys (8 years old) said that the presented items were “coins and notes”. All the children, when asked about what they could do with the money, answered that it is used “to pay for something” or “to buy things”. Two older boys and one girl (all 8 years old) also stated that money could be “saved”.

In the recognition task, the coins posed a greater difficulty than the notes for the children. Correct coin recognition was obtained only in 88% of the cases and correct note recognition was made in 99% of the cases. The most difficult denominations to recognize were the smallest: 5 gr (only 66% correct recognition), 2 gr (79% correct recognition), and 1 gr (83%correct recognition). All mistakes in the recognition task were made by children that were 6 and 7 years of age; all the 8-year-old children performed this task faultlessly. The task of distinguishing Polish money from other objects was solved correctly only by 41% of the participants. The wrong answers were given by children of all ages. A large number of children (54%) pointed to the old Polish money (withdrawn from circulation after the denomination in Poland) as the current money. Furthermore, 21% of the children indicated that Monopoly money is the current Polish currency that could be used in shops, and 12% of the participants stated that some of the smallest denominations of Polish coins (gr) could not be used to pay for things in shops. Only 71% of the children indicated the correct coin when they were asked to choose the coin that would let them buy the largest portion of sweets (2PLN). Twenty-five percent of the children incorrectly stated that the 50 gr coin possessed the biggest purchasing power. All the mistakes in this task were made by children of 6 and 7 years of age; all 8-year-old children performed this task correctly. Seventy-five percent of the participants did well in the task involving changing 5PLN, and 71% successfully calculated the exact amount (7PLN 21 gr). Most mistakes were made by 6- and 7-year-old children; only one 8-year-old made a mistake in calculating 7PLN 21 gr. The task that posed the most difficulties for the children was calculating the change when the experimenter had to pay 7PLN 21 gr but provided a 10PLN note. Only 21% of the participants gave the correct change to the experimenter. The younger children (6 and 7 years of age) often failed to understand the notion of giving change (41% of the younger children), while the older children had problems with correctly performing their calculations (although they were familiar with the notion of giving change).

The results of the pre-test study indicated that children between 6 and 8 years of age have some, but not full knowledge of the instrumental function of money. These findings are consistent with the results obtained by Kupisiewicz (2004), and show that children (6- to 8-year-olds) know what money is used for but often made serious mistakes when asked to calculate prices, distinguish money from other objects, give change, and when asked about the purchasing power of money.

## Study 1

The main aim of Study 1 was to investigate whether money activation had any bearing on the length of time for which children persevered with a difficult task. The participants were 6-year-old children and were expected to know what money looks like but were not required to demonstrate the proper use of money in transactions (Berti and Bombi, 1988; Kupisiewicz, 2004, pre-test study described above). The following prediction was made:

**Hypothesis 1:** Money activation prompts children to persevere with a difficult task for longer.

### Materials and Methods

#### Participants

Sixty-one preschoolers (all 6 years old; 34 girls and 27 boys) took part in this study. The experiment was carried out in two nursery schools in Warsaw, Poland, with the consent of the headmasters and teachers. The experiment was conducted in accordance with the Declaration of Helsinki and was approved by the Ethics Board of the Faculty of Psychology at the University of Warsaw. The parents of all the children provided a written informed consent for their children’s participation in the experiment.

#### Procedure

The experiment consisted of two stages: experimental manipulation and a puzzle-solving task. All the children were assessed individually. The children were randomly assigned to either the experimental condition (*n* = 30) or the control condition (*n* = 31). At the beginning of the study, the experimenter showed the participant a hat containing 10 items; in the experimental condition, there were five coins and five bills, whereas in the control condition, there were five round pawns and five playing cards. The experimenter then asked the participant to select three items from the hat, then another five items, and finally the last two items. After this, the child was asked to count all the items that had been drawn from the hat. In the second stage, the participants tackled seven-piece, wooden puzzles which required them to fit the seven differently shaped pieces exactly inside a rectangular frame drawn on a sheet of paper. There was only one solution to each puzzle. The task was selected so that it would appear easy but was, in fact, difficult for the children. After explaining the task, the experimenter told the participant that they would leave the room so that the participant would not be disturbed whilst working on the puzzles and showed the participant a button-operated bell (on a box in front of the participant) that could be used to summon the experimenter if any help was required with the task or to notify the experimenter of when the participant had finished the task. On hearing the bell, the experimenter returned to the room to help the child or thank him or her for their participation in the experiment. The maximum time for which the children were left to work alone was 15 min, if the child had not finished the task or asked for help at the end of that period, the experimenter returned to the room and finished the experiment.

### Results

The dependent variable was the time the child spent on trying to solve the task without asking for any help. Hypothesis 1 predicted that children from the experimental group would persevere for longer before asking for any help compared to the children from the control group. We were also interested in whether the child solved the puzzles unaided. As expected, the task proved to be difficult for the children and only 7 out of 61 participants successfully solved the puzzles unaided. The average time spent on trying to solve the task before asking for help was 90.20 seconds (*SD* = 106.94 s). Children in the experimental condition spent a longer time trying to complete the puzzles than the participants in the control group, [**Table 1**; *F*(1,59) = 9.452; *p* < 0.005]. This result supports Hypothesis 1, which suggests that money activation prompts children to persevere with difficult tasks. Moreover, 23.3% of the children in the experimental group completed the task, whereas none of the children in the control group managed to do so (**Table 1**). This difference in effectiveness was significant [*χ^2^*(1) = 8.171, *p* < 0.005]. There were no gender differences between the time spent solving the task [*t*(58) = -0.494, *p* = 0.623] and the effectiveness [*χ^2^*(1) = 0.006, *p* = 1].

**Table 1:** Perseverance and effectiveness of children in the control and experimental condition.

	Perseverance (time spent on task-solving)	Effectiveness (percentage of children who successfully solved all the puzzles)
Control condition	51.83 s (*SD* = 63.33)	0
Experimental condition	131.24 s (*SD* = 127.62)	23.3

## Study 2

The objective of Study 2 was to examine how activating the concept of money affected children’s choices about the timing of a reward. The participants were children aged 6–8 years who were expected to be on the second, general level of economic socialization (Furnham and Argyle, 1998), thus, to have some understanding of economic ideas in isolation but lack of full appreciation of the concept of money and still be on the concrete operations stage. Taking into account the results of Vohs et al. (2006, 2008), the following was predicted:

**Hypothesis 2:** In children aged 6–8 years, money activation increases the probability of children opting to delay gratification.

### Materials and Methods

#### Participants

To ensure that our analyses only refer to children who do not possess a full understanding of the instrumental functions of money, we decided to exclusively include children up to the age of 8 in the analysis. According to Kupisiewicz (2004) and our pre-test study, Polish children under the age of 8 reveal significant gaps in knowledge about money. Forty-six children (29 girls and 17 boys) aged 6–8 years (*M* = 7.38 years, *SD* = 0.62, Mdn. = 7.00 years) took part in Study 2. The experiment was conducted in accordance with the Declaration of Helsinki and received the approval of the Ethics Board of the Faculty of Psychology at the University of Warsaw in Poland. The parents of all the children provided a written informed consent for their children’s participation in the experiment.

#### Procedure

The experiment consisted of two stages: manipulation and decisions concerning delaying gratification. The first stage was conducted with small groups of six to eight children. The groups were randomly assigned to the experimental condition (*n* = 20, *M* age = 7.27 years, *SD* = 0.68, Mdn. age = 7.00 years) or to the control condition (*n* = 26, *M* age = 7.46 years, *SD* = 0.56, Mdn. age = 7.50 years). First, the experimenter showed 10 items to the participants and asked several questions about them (e.g., *What are these items?*; *How many can you see?*). In the experimental condition, the set of items was comprised of five coins and five notes; in the control condition, it included five round pawns and five playing cards. The children were then handed sheets of paper and crayons and asked to draw something associated with the items that were presented to them. An analysis of their drawings showed that all the children in the experimental group drew coins or notes, whereas the children in the control group drew pictures related to various games (playing cards, computer games, or board games), with the exception of one boy who drew coins. This boy was excluded from the statistical analyses. After the picture-drawing activity, the children were led into another room, one by one (in the order in which they finished their drawings), to choose a reward for their drawings (the second stage). The children were asked to choose between two options: (1) choose one sticker and take it away with them now, or (2) wait until tomorrow to get two stickers where the experimenter told the child that he or she would have more stickers later, which would mean leaving the room at that specific time with no stickers at all. The following day, all the children who had decided to delay the gratification were given the opportunity to choose from and take two stickers away with them.

### Results

The dependent variable was the binary decision about the timing of gratification. Hypothesis 2 predicted that children from the experimental group would be more likely to decide to wait 1 day in order to get an extra sticker than children from the control group. This prediction was fulfilled and 85% of the children in the experimental group decided to wait 1 day longer to get a bigger gratification, whereas only 53.8% of the children in the control group decided to do so [*χ^2^*(1) = 4.993 *p* < 0.03]. This result supports Hypothesis 2 and suggests that money activation increases the probability of deciding to delay gratification in order to increase the magnitude of the reward. There was no gender effect on the decisions concerning delaying gratification [*χ^2^*(1) = 1.012 *p* = 0.32].

## Discussion

The two experiments reported herein support the hypotheses that money activation (1) influences children’s perseverance and effectiveness in difficult individual tasks, and (2) increases the probability of children choosing to delay gratification in order to obtain a larger reward. This showed that the children participating in our studies reacted to money priming in a similar way to the adults and undergraduates who took part in the experiments reported by various researchers (Mogilner, 2010; Boucher and Kofos, 2012; Vohs, 2015). During the review process of the present paper, Gąsiorowska et al. (2016) published their research which also demonstrated that the activation of the money concept in children increased the effort that children put into the completion of a difficult task. Such results further confirmed and strengthened the conclusions of our first experiment. In our research, the money concept evoked a focus in the children on the individual and difficult task to be completed by them, which led to an improvement in their performance. It can be assumed that the mere idea of money has enhanced the self-control needed to confront challenges and attain important outcomes. It is also worth noting that the money activation in our experiments caused the children to put more effort into solving difficult tasks without expecting any help from others (e.g., from the experimenter). This result confirms the conclusions of Gąsiorowska et al. (2016) that handling money inhibits communal behavior in children.

The aim of the study was to conduct research among children who, due to their age (Berti and Bombi, 1988; Kupisiewicz, 2004), do not yet understand the instrumental functions of money fully. This assumption was confirmed in the pre-test study, the results of which demonstrated that children between the age of 6 and 8 often make serious mistakes when dealing with tasks involving calculating and distinguishing money. The participants in both experiments were on the second, general stage of economic socialization and were incapable of understanding market mechanisms or using money correctly in transactions. Given this fact and following the outcome of the study conducted by Gąsiorowska et al. (2012), it can be concluded that the reactions of our study participants to money mainly reflect the activation of the symbolic function of money. Children reacted to money priming similarly to adults, which means that they must possess some understanding of money. Due to the fact that children between the age of 6 and 8 do not fully comprehend the instrumental function of money, it is probable that some emotional connotations (symbolic meanings) with money were responsible for the effects of the money priming. Our results support the hypothesis (Gąsiorowska et al., 2012) that the symbolic function of money is more primal than its instrumental function and probably develops earlier on in life.

Previous research (Gąsiorowska et al., 2012) has showed that there is a “dark” side to the symbolic function of money by providing some evidence that the activation of the money concept resulted in children being less willing to provide help and more prone to making more selfish choices. Our experiments, using a design similar to that employed by Vohs et al. (2006, 2008) in research conducted on adults, demonstrated that the activation of the money concept also has positive effects, as it is associated with a greater perseverance in children, that is, they were willing to work alone on a difficult task for longer, and also increases the likelihood that they would choose to delay gratification. However, it is worth noting that the focus on solving difficult tasks without asking for help (after money activation) is probably associated with reduced communal behavior. Therefore, this effect may be considered as positive (increased task-solving efficiency) on the one hand, but negative (decreased communal behavior), on the other.

Our research supports Gąsiorowska et al. (2012) results demonstrating that children on the early stage of economic socialization who do not possess a comprehensive understanding of the instrumental functions of money react to the activation of the symbolic meaning of money. The researchers (Gąsiorowska et al., 2012) hypothesized that this possibly implies that the symbolic meaning of money develops somewhat independently from the understanding of its instrumental function and is more strongly related to social learning than to cognitive development (Piaget and Inhelder, 1972). Future research is needed to draw out clearer ideas about the relationship between the development of the symbolic and instrumental understanding of money in children. It would also be worth investigating how parental attitudes to money influence their children’s symbolic associations with money.

The present studies have certain limitations. The first is that economic socialization processes are rapidly progressing among children aged between 6 and 8. Although children at this age do not possess a comprehensive understanding of the instrumental meaning of money, each child develops at his or her own pace and possesses a different level of knowledge. Therefore, it would be worthwhile carrying out a study in which the actual knowledge of the instrumental functions of money would be controlled. Moreover, research on children who are on the first stage of economic socialization (under the age of 5) could enrich the conclusions of the present research further.

Some recently published, failed replications of Caruso et al.’s (2013) money priming experiments (Rohrer et al., 2015) could be argued to constitute the second limitation of the present studies. However, Vohs (2015) provides extensive, possible explanations of Rohrer et al. (2015) results in her research, claiming that it could be the effect of a sampling issue. The failed replication of Caruso et al.’s (2013) study is probably due to the differences in the meaning attached to money by the participants who took part in those studies. It should also be noted that the results of the experiments presented in this article are in line with the results obtained by various researchers in a diverse range of locations (see: Vohs, 2015), and show that money priming can indeed influence people’s behaviors.

## Author Contributions

AT and KS planned the research, designed the studies, and analyzed the data. AT wrote the manuscript along with inputs from KS during revisions.

## Conflict of Interest Statement

The authors declare that the research was conducted in the absence of any commercial or financial relationships that could be construed as a potential conflict of interest.
